# Perspectives and Experiences on eHealth Solutions for Coping With Chronic Pain: Qualitative Study Among Older People Living With Chronic Pain

**DOI:** 10.2196/57196

**Published:** 2024-09-05

**Authors:** Annalisa De Lucia, Valeria Donisi, Ilenia Pasini, Enrico Polati, Lidia Del Piccolo, Vittorio Schweiger, Cinzia Perlini

**Affiliations:** 1 Section of Clinical Psychology Department of Neuroscience, Biomedicine and Movement Sciences University of Verona Verona Italy; 2 Anesthesiology, Intensive Care and Pain Therapy Center Department of Surgery, Dentistry, Paediatrics and Gynaecology University of Verona Verona Italy

**Keywords:** older adults, qualitative method, pain, coping strategies, eHealth, pain management, mobile phone

## Abstract

**Background:**

Chronic noncancer pain (CNCP) is a major health issue among the older population, affecting multiple aspects of individual functioning. Recently, the use of eHealth solutions has been proposed in supporting chronic pain self-management even among older adults, although some barriers have emerged. Few qualitative studies, with none conducted in Mediterranean countries, have explored older people’s experiences and perceptions regarding the types of strategies used to cope with chronic pain and eHealth tools for chronic pain management.

**Objective:**

This study’s objectives were to explore the perspectives and experiences of older adults regarding the coping strategies used to manage chronic pain, the use of digital technologies in everyday life, and the potentiality and barriers in using those technologies for health and pain management.

**Methods:**

A multimethod approach (ie, self-report questionnaires and a semistructured interview) has been adopted targeting older adults (ie, those who are aged 65 to 80 years and presenting different types of CNCP) who are attending a pain therapy center in Italy. Qualitative answers were analyzed using thematic analysis.

**Results:**

Overall, participants reported using a variety of pain coping strategies; however, they showed an attitude of resignation to their CNCP condition. Nearly 70% (12/18) of the interviewees referred to using digital technologies for purposes related to health and pain management, mostly involving very basic management activities. The participants’ opinions on the useful functions that need to be incorporated in eHealth tools for chronic pain management have been categorized into four themes: (1) *specific pain self-management skills*, (2) *support in organizing various health-related aspects*, (3) *sharing experiences with other*s, and (4) *increasing pain-related personal knowledge*. Conversely, the following potential barriers to adopting eHealth tools emerged: (1) *computer illiteracy*, (2) *negative effects or risks*, (3) *impersonal interaction*, and (4) *physical limitations*.

**Conclusions:**

The use of eHealth solutions still seems low, often being accompanied by a perceived lack of digital skills or attitude among a sample of older adults from Italy with CNCP. Before introducing innovative eHealth solutions, it would be of primary importance to take action to enhance, on the one hand, self-efficacy in pain management and, on the other, the digital literacy level among older people.

## Introduction

### Background

Chronic pain (ie, pain lasting for >3 months) [1] is a major global health issue, affecting approximately 20% of people worldwide [2]. The prevalence of chronic pain generally increases with age, and it is estimated to be higher in adults aged ≥65 years, with a substantial negative impact on their physical, psychological, and social functioning [3-5]. Because of the several physiological age-related changes and frequent pathological comorbidities, effective treatment of chronic pain can be challenging in the older population [6]. Furthermore, in addition to the reluctance of older people to seek or accept medical help, the common belief among older adults and health care providers that pain is a normal part of aging may partially explain why chronic pain is often underestimated and underreported [7]. In this regard, a few studies showed that older adults tend to adopt a stoic attitude while experiencing pain, most often preferring to use self-reliance–based coping strategies, although they are not always effective [7,8]. Several studies have explored the life experiences and needs of older adults with chronic pain as well as the strategies they use to cope with it [9-11]. These studies also aim to promote more effective and tailored pain self-management interventions [8,11,12].

In recent years, particularly in the wake of the COVID-19 pandemic, there has been a massive increase in the adoption of eHealth solutions to ensure the continuity and accessibility of care [13]. Preliminary evidence supports the potential of these solutions in pain assessment, diagnosis, and treatment [14]. However, older adults, especially subgroups aged 75 to 84 years and ≥85 years, still seem to be underrepresented in this field of research [15]. There is, indeed, evidence that older people are more likely to experience digital exclusion, even in research settings, due to age-related negative stereotypes that depict them as a homogeneous group of technologically illiterate users [16-18]. This often results in a lack of consideration of their needs and preferences from the earliest stages of the eHealth solutions design process, with the risk that the proposed tools do not fully match the real needs of end users and are thereby weak in terms of feasibility and acceptability for older people [19-22].

Although it represents a growing area of interest, to date, to the best of our knowledge, only a few qualitative studies have explored the specific experiences and perspectives of older adults on the use of eHealth solutions in the context of coping with chronic pain. Among the recent studies, Bhattarai et al [23] carried out a qualitative study aimed at evaluating older people’s views and experiences of using a smartphone app, with the sole focus on chronic arthritic pain management. In addition, O’Reilly et al [20] identified barriers and specific needs of both middle-aged and older adults (mean age of 61.3, SD 7.7 y) regarding the use of eHealth solutions for chronic pain self-management. Further qualitative approaches have been introduced with the specific aim of developing eHealth tools and often targeting a specific population of patients with chronic pain (those with osteoarthritis [24], older adults with obesity and experiencing chronic pain [25], those with chronic back pain [26], those with chronic pain in comorbidity with cognitive decline [27], and those with chronic musculoskeletal pain [28]).

To the best of our knowledge, none of the aforementioned studies were carried out in a Mediterranean country, such as Italy.

It should be indeed considered that values; norms; and, in general, the cultural framework can significantly affect people’s life experiences and health-related issues, including the ways in which pain is perceived and managed [11,29]. For example, older adults living in Mediterranean countries seem to value and rely more strongly on close family support in managing well-being, compared with those living in Nordic countries, for whom values such as autonomy and a tendency to rely on formal caregivers outside the immediate family entourage prevail [30]. Concurrently, the use of technology may vary according to the sociocultural context [31]. Interestingly, as shown in recent qualitative research about internet use among people aged ≥65 years during the COVID-19 pandemic [32], many Italian older people tended to seek technological help from close family members such as children or grandchildren. This propensity to ask for help or to totally delegate the execution of activities using technological devices, for example, may at least partly impact the degree to which older people are motivated and willing to enhance their digital skills for independent use of such devices.

### Objectives

This study aims to integrate previous research by exploring perspectives and experiences regarding eHealth solutions for coping with chronic pain among older adults with different types of chronic noncancer pain (CNCP) in the context of an Italian center for pain management. Specifically, this paper aims to explore the perspectives of this population regarding (1) the types of strategies used by older adults to cope with chronic pain and the related perceived effectiveness, (2) the types of digital technologies adopted in everyday life and the purposes of their use, (3) the use of digital solutions for health and pain management and older adults’ experience with those solutions, and (4) the potential useful functions to be included in eHealth solutions for chronic pain self-management and the barriers to use such tools.

## Methods

### Ethical Considerations

The study was approved by the local clinical research committee of the Verona University Hospital (Registro del Dolore protocol, ID 1751CESC) and was conducted in accordance with the principles of the Declaration of Helsinki. Written informed consent was obtained from all participants. The participants did not receive any form of compensation. The collected data have been anonymized to safeguard participants information.

### Study Design

This study used a multimethod approach: self-report questionnaires and semistructured interviews.

### Participants and Setting

A purposive sample of community-dwelling older adults living with chronic pain was recruited from the pain therapy unit of the Verona University Hospital, Italy. This highly specialized center offers clinical activities for the diagnosis and pharmacological and nonpharmacological treatment of all forms of pain (eg, low back and osteomuscular pain, fibromyalgia syndrome, maxillofacial complex pain forms, headaches, neuropathic pain, complex regional pain syndromes, and district pain). It is mainly attended by people whose pain is difficult to manage in primary care settings and who often undergo several types of treatments unsuccessfully.

Self-report questionnaires and a semistructured interview were administered to all patients evaluated at the outpatient center who met the following inclusion criteria: age range between 65 and 80 years; having a clinical diagnosis of CNCP (lasting for at least 3 months), as defined by the referring physicians according to the clinical records; being an Italian speaker; and being able to answer interviews and questionnaires as established by the referring physicians.

Available patients were approached from the end of September to the beginning of December 2023 at the pain therapy unit, and recruitment continued until data saturation in the interviews was reached [33].

### Measures

Sociodemographic (ie, sex, age, educational level, and civil status) and clinical data (ie, type of CNCP diagnosis, pain intensity, and duration) were collected immediately before starting the administration of the questionnaires and interviews. The specific diagnosis of CNCP was made by the referring physicians based on the widely accepted temporal criterion by which persistent pain is any pain lasting >3 months [34]. Pain intensity on average over the last week was measured by verbally asking participants to rate their pain on a scale from 0 (ie, no pain) to 10 (ie, the worst pain imaginable).

Participants were asked to fill out the following self-reported questionnaires: the Psychological Well-Being Questionnaire (Ben-SSC [35]) and the Chronic Pain Coping Inventory-Italian version (CPCI-I [36]).

The Ben-SSC [35] is a validated 37-item questionnaire specifically constructed to assess psychological well-being in both the adult and older Italian populations. It is inspired by the eudaimonic perspective of well-being [37], covering 3 different dimensions: personal life satisfaction, sense of autonomy and self-efficacy, and emotion-regulating skills. The participants are asked to indicate their degree of agreement with each item on a 4-point Likert scale, ranging between 1 (not at all) and 4 (always). The Ben-SSC provides a total well-being score, obtained by summing the score for each of the 37 items, where higher scores indicate higher levels of psychological well-being. The 3 subscale scores are calculated by dividing the sum of the items’ scores by the number of items in that subscale. The Ben-SSC demonstrated good reliability (Cronbach α=0.91) [35].

The Italian version of the 42-item CPCI-I [36] is a self-report questionnaire that focuses on asking the users to rate the frequency of use of cognitive and behavioral pain coping strategies during the previous week. The strategies are categorized into the following 8 subscales: guarding (avoiding or restricting the use of a body part or movement or specific activities), resting (eg, sitting and laying), asking for assistance (asking someone for help with a task), relaxation (engaging in specific relaxation exercises to reduce muscle tension, eg, slow, deep breathing), task persistence (eg, ignoring pain and carrying out an activity despite the pain), exercise or stretch (doing specific muscle-strengthening or stretching exercises), seeking social support (talking with a significant person to receive support when one experiences pain), and coping self-statements (encouraging oneself through positive thoughts about the painful condition). Each item is scored on a 0 to 8 points scale, where higher scores indicate a greater use of coping strategies. Subscale scores are obtained by dividing the sum of the items’ scores by the number of items in that subscale. The CPCI-I proved to have a good factorial structure and psychometric properties similar to the original and adapted versions [36].

The face-to-face semistructured interviews were conducted by ADL at the pain therapy unit. Interviews were based on an interview guide developed by the multidisciplinary research team (Multimedia Appendix 1). The included questions mainly explored the following aspects: the pain coping strategies perceived as most effective among those indicated in the CPCI-I and potential additional helpful coping strategies (ie, not included in the CPCI-I coping categories), the type of digital technologies used and their purposes of use, the experiences regarding health-related purposes of use for digital technologies, and the potential useful functions and barriers to use eHealth solutions for chronic pain management.

### Data Analysis

Descriptive statistics were used to summarize the sociodemographic and clinical characteristics of the sample and questionnaire scores.

For data collected qualitatively (ie, semistructured interview), for each theme, we reported examples (the participant ID code to which the quote corresponds is provided in brackets) and the number of quotes related to that theme. All interviews were transcribed “verbatim” in real time and anonymized by alphanumeric codes. A reflexive thematic analysis was used to identify and analyze patterns within the collected qualitative data following the 6 steps proposed by Braun and Clarke [38-40]. More specifically, an inductive and epistemologically constructionist approach to understanding the data was adopted to better capture the meaning and meaningfulness attributed by the participants. Consequently, apart from the recurrence of certain themes within the data set, major importance in the coding process was placed on the significance or relevance of potential themes to the research questions. After familiarizing ourselves with the data by reading the transcripts several times and noting down initial ideas (step 1), the data were initially coded (step 2) and then sorted into potential themes and subthemes (step 3). Next, these preliminary themes were compared and refined repeatedly (step 4) until coherent and meaningful patterns were obtained both at the level of individual themes and of the data set as a whole (step 5). Finally, a concise, coherent, and nonrepetitive story of the data was provided, and representative quotes were provided to exemplify the themes selected (step 6). Data analysis was performed by 2 researchers (ADL and IP) independently, and a third researcher was involved in the discussion when disagreement emerged (VD).

Diverse steps have been used to guarantee the rigor of qualitative methods [41]. More specifically, credibility was ensured by investing an adequate period in the research setting and interacting with the participants (ie, prolonged engagement) and using investigator triangulation. The triangulation, along with the involvement of a researcher not directly engaged in the data analysis and collection (LDP), allowed us to ensure consistency, repeatability, and confirmability of the methods.

## Results

### Sociodemographic and Clinical Characteristics of the Sample

A total of 23 participants were considered eligible according to the inclusion criteria and approached during the recruitment period. Of these 23 participants, 3 (13%) refused to participate in the study, 1 (4%) discontinued completing the questionnaires due to time constraints, and 1 (4%) was excluded because of substantial hearing impairment that prevented an accurate understanding of the interview questions. However, data saturation (ie, recurrence of themes when no new information emerged) was achieved after interviews with 18 participants, thus not proceeding with the recruitment. The numerosity results are in line with the sample sizes reported in previously published qualitative exploratory research [10,11,42].

On average, the completion of the questionnaires and interviews took about 40 minutes, ranging from 30 to 50 minutes. All questions included in the questionnaires and the interviews were answered by the participants.

Table 1 describes the participants’ sociodemographic and clinical characteristics. The average age of the participants was 72.7 (SD 5.2; range 65-79) years. Most of them were female (14/18, 78%) and married (12/18, 67%), with a primary or middle school level of education (14/18, 78%).

**Table 1:** Participants’ sociodemographic and clinical characteristics.

ID	Sex	Age (years)	Educational level	Civil status	Chronic pain condition	Pain intensity (0-10)	Pain duration
1	Female	79	Primary school	Married	L5 radiculopathy (persistent canalar stenosis) on the right side	10	7 to 8 years
2	Female	75	Primary school	Married	Fibromyalgia	8	Lifelong
3	Female	75	Middle school	Married	Low back pain on the right side	5.5	4 to 5 years
4	Male	78	Primary school	Married	Postherpetic neuralgia	10	3 years and 7 months
5	Female	66	Middle school	Married	Low back pain	7	About 8 months
6	Male	74	Middle school	Married	Low back pain	5	Pain for several years that has worsened for about 20 days
7	Female	73	Middle school	Married	Radiculopathy and rheumatoid arthritis	8	18 years
8	Female	79	Primary school	Widower	Neck pain	8	A few years
9	Male	75	Middle school	Married	Postherpetic neuralgia	3	1 year
10	Female	65	University	Married	Fibromyalgia and Sjögren syndrome	4.5	10-year diagnosis but lifelong pain
11	Female	65	High school	Married	Postherpetic neuralgia	10	4 years
12	Female	75	Middle school	Married	Low back pain	10	36 years
13	Male	75	Primary school	Celibate	Neuropathic pain	10	33 years
14	Female	74	Primary school	Divorced	Low back pain	10	20 years
15	Female	79	Primary school	Widower	Fibromyalgia	9	Lifelong
16	Female	70	University	Widower	Fibromyalgia	8	Diagnosed in 2018 but pain for almost 20 years
17	Female	66	High school	Divorced	Chronic migraine	7	46 years
18	Female	65	Middle school	Married	Lumbosciatalgia and neck pain	7	8.5 years

Participants reported a wide range of CNCP conditions, among which the most frequent were low back pain, fibromyalgia, and postherpetic neuralgia, with an average pain intensity level of 8.1 (0 to 10 scale). The duration of pain ranged between 8 months and “lifelong,” with 50% (9/18) of participants experiencing pain for >20 years.

### Psychological Well-Being

The total mean score of the Ben-SSC was 109.2 (SD 17.2), indicating a medium level of perceived psychological well-being. The mean scores obtained in each subscale of the Ben-SCC also reflect a medium level regarding the specific constructs of personal satisfaction (mean 32.3, SD 8.1; possible range 11-44), perceived self-efficacy and sense of autonomy (mean 25.7, SD 5.2; possible range 9-36), and emotion regulation skills (mean 30.8, SD 3.6; possible range 10-40).

### Coping Strategies to Manage Chronic Pain

The most adopted strategies to cope with pain, as assessed by the CPCI-I, were coping self-statements (mean 4.3, SD 1.6), resting (mean 4, SD 1.9), task persistence (mean 3.9, SD 1.6), and guarding (mean 3.7, SD 1.9). The least used strategies were relaxation (mean 2, SD 1.4) and exercise or stretch (mean 2.3, SD 1.7; Table 2).

**Table 2:** Total sample mean scores for each of the Chronic Pain Coping Inventory-Italian version subscales (score range for each subscale: 0-8).

Coping strategies	Score, mean (SD)
Coping self-statements	4.3 (1.6)
Resting	4 (1.9)
Task persistence	3.9 (1.6)
Guarding	3.7 (1.9)
Seeking social support	3.3 (2)
Asking for assistance	3.2 (1.7)
Exercise or stretch	2.3 (1.7)
Relaxation	2 (1.4)

On the basis of the participants’ responses to the interview questions, among the strategies indicated in the CPCI-I, those perceived to be most effective were resting (6/18, 33%), followed by exercise or stretch (3/18, 17%), task persistence (2/18, 11%), guarding (2/18, 11%), relaxation (1/18, 6%), and seeking social support (1/18, 6%). Of the 18 participants, 1 (6%) participant indicated >1 strategy as the most effective, and 4 (22%) participants reported that none of these strategies were effective. The average perceived efficacy level in terms of pain reduction of the referred strategies was 5.9 (SD 3.4; 0 to 10 scale). As for the scores assigned to each CPCI-I strategy, the values ranged from 7 to 10 for exercise or stretch, 1 to 10 for resting, while the remaining strategies—mentioned less frequently as being effective—were assigned the following scores: 3 and 5.5. for task persistence, 4 and 7 for guarding, 3 for relaxation and 6 for seeking social support.

Additional coping strategies (ie, not evaluated through the CPCI-I) have been reported by participants during the interview. Quotes regarding the additional coping strategies were grouped into the following categories:

1. Body treatments with home remedies: some participants (n=6 quotes) reported resorting to home-based informal strategies to relieve pain, including massages, warm or cold modalities, and salt and water baths, such as in the following case:

I take a bath with water and salt. Also, I warm my leg with my phono, and I have to warm it slowly or it hurts even more.

ID 13

2. Medications: for 4 participants (n=4 quotes), taking medications, for example, anti-inflammatory drugs, have been described as an effective, and sometimes as the only possible, strategy:

Besides resting, there is no other strategy, only anti-inflammatories.

ID 2

3. Spirituality, such as prayer: 2 respondents (n=2 quotes) declared that they seek hope and comfort in faith:

I pray a lot; prayer calms me down... in addition to reciting the formulas, I speak within myself freely, from the heart.

ID 7

4. Adjusting daily rhythms to pain: this strategy reflects the tendency to perceive pain as unavoidable and the consequent need to change certain lifestyle habits to continue one’s daily activities to the best of one’s capabilities. Specifically, to best pursue his professional activity, a participant said the following:

I adopted a system, I sleep from 8 p.m. to midnight, the pain is not so intense... afterward I take the pills and sleep maybe half an hour until morning... I totally changed my routine, but I had to do it.

ID 6

5. Resignation: an attitude of distrust of health services emerged from 1 participant, indicating a reduction in expectations regarding the actual help provided by the health care system for the treatment of pain:

I feel little help from institutions, doctors, professionals, there is no listening. We are numbers, when I come to a new institution my motto is don’t expect anything.

ID 10

### Type of Digital Technologies Used by the Participants and Purposes of Use

All the interviewees (18/18, 100%) reported owning a mobile phone and using it independently, while only 3 (17%) of them referred to using other technological devices, such as computers, tablets, and smartwatches.

As for the general purposes of use in daily life, all participants indicated that they use digital technology tools *to keep up connections with others* (n=18 quotes), and more than half of them also use these tools *for leisure activities* (n=14 quotes):

The mobile phone for me is essential for communicating with my daughters and grandchildren who don’t live very close to my home... either with calls or messages or even video calls.

ID 14

I usually use my mobile phone to play cards to while away the time....

ID 4

I like visiting websites of catechesis and prayer, listening, watching videos...

ID 11

Of the 18 participants, 6 (33%) declared *other daily life uses* of digital solutions, such as web banking and shopping. (n=7 quotes):

Other things that I do are for example accessing my bank account, making payments and bank transfers....

ID 14

Only 1 (6%) of the 18 participant spontaneously reported engaging in health-related activities, specifically searching the internet for information related to one’s medical condition or any new pharmacological therapies being undertaken (ID 12).

### Experiences of Using Technological Solutions for Health-Related and Pain-Related Purposes

When participants were explicitly asked whether they use such devices for purposes related to health management (including pain conditions), nearly 70% (12/18) of the interviewees answered affirmative, while the other participants declared that they do not directly use eHealth solutions (even if in some of these cases, they delegate the use of these tools to informal caregivers).

The quotes reported by affirmative responders have been categorized into the following themes:

1. *Booking medical visits or tests* (n=11 quotes):

I often book medical examinations on the internet or call the medical service directly.

ID 18

2. *Searching health information* (n=8 quotes):

When I am prescribed new drugs, I go and look online to find out what it is.

ID 12

3. *Managing pharmaceutical prescriptions* (n=6 quotes):

Sometimes I use this app of the National Health System, that allows you to receive and manage medical prescriptions.

ID 11

4. *Accessing the personal electronic health record* (n=4 quotes):

I always use the Electronic Health Record, for example, I check if the doctors have uploaded the reports, I keep track of all the medical examinations...

ID 16

5. *Purchasing medicines on the web*: Of 18 participants, 2 (11%) stated ordering and purchasing medications on the web (n=2 quotes):

I find it good to order medication on the internet, it is a fast method, the medicines arrive directly at home in no time.

ID 18

As for the participants’ current use experiences of digital devices for health-related purposes, the reported quotes have been categorized into 3 themes, with 1 concerning the quantity and 2 regarding the quality (respectively, positive or negative) of such experiences.

1. *Limited to few activities*: for 5 (28%) of the 18 participants, the use of digital technologies (mainly mobile phones) is limited to a few activities related to their health management (eg, booking medical visits and tests and downloading clinical reports) or is not very frequent (n=5 quotes):

I don’t know many things, but I am able to do the things that are essential to me [for healthcare].

ID 8

2. *Positive and helpful experience* refers to fulfilling experiences related to the use of digital technologies in health care, as reported by 4 (22%) of the 18 participants, with the following example being 1 of the 4 quotes:

Technology is a great help and support in managing several aspects of my health and my painful condition.

ID 10

3. *Negative experience*, which was reported by 3 (17%) of the 18 participants, included answers related to “external support/delegating to others” (n=2 quotes) and “unsatisfactory” in relation to malfunctions (n=1 quote):

There are some things that I can’t do, for example checking emails, and therefore I ask my neighbor, a very nice lady, to do these things for me.

ID 15

Some systems, such as the Electronic Health Record, are potentially useful, but they must work properly! But it rarely happens, there is always a problem.

ID 16

Specifically, for the first subtheme, despite the independent use of digital devices for some basic health-related activities (eg, making a phone call to book a medical visit), a few of the interviewees (3/12, 25%) highlighted the need for assistance or to delegate the execution of more complex operations to others.

### Potential Useful Functions of eHealth Tools for Chronic Pain Self-Management

According to 10 (56%) of the 18 interviewees, adopting eHealth tools specifically designed for pain management might be useful.

Regarding the potential useful functions of possible new eHealth tools for pain management, we identified the following four themes: (1) s*pecific pain self-management skills* (n=7 quotes), (2) *support in organizing various health-related aspects* (n=2 quotes), (3) *sharing experiences with others* (n=2 quotes), and (4) *increasing pain-related personal knowledge* (n=2 quotes). Of the 18 participants, 3 (17%) reported having no specific ideas about possible functions to be included in such tools, although they expressed themselves favorably about the usefulness of possible eHealth solutions.

The first theme includes several strategies that participants find helpful for achieving better self-management of pain to be delivered, for example, through text, audio, or video files explaining good practices, physical exercises or stretching, and relaxation or meditation practices. Indeed, just over half of the interviewees (6/10, 60%) expressed the need to strengthen their pain self-management skills through targeted content relating to both the physical or biological and psychological or educational fields. For example, a participant said the following:

Looking for an alternative to medication... for example, when I wake up in the morning, I feel the need to stretch my muscles... so an alternative might be videos showing specific exercises or relaxing therapies.

ID 12

In addition, some participants argued for the usefulness of providing professionals with different backgrounds and the possibility to select the proposed activities according to one’s interests. This is to emphasize the necessity of interacting with a range of experts and choosing content that is not “standard” and applicable to all but rather unique depending on the specific requirements of everyone:

It would be helpful to include the names of experts with different backgrounds, not only health care, but also theologians or meditation teachers to have moments of reflection.

ID 11

I would include both “random” and “user-selected” activities, because I would be interested, for example, in videos of professionals with a spiritual background but others might prefer a more medical approach.

ID 15

The second theme focused on the support provided by digital tools in organizing various health-related aspects. Some participants stated it could be helpful to receive automatic notifications on their smartphones to prevent forgetfulness regarding scheduled appointments, as explained in the following case:

First of all, a reminder if I have to do something, for example, appointment reminders... for example, the day before the visit it comes up on my cell phone screen that tomorrow I have to undergo that medical examination.

ID 1

Furthermore, the need for digital systems that allow medical appointments to be booked quickly and easily was highlighted:

A program that allows me to quickly book visits.

ID 5

The third theme addresses the need for social support and the desire to share experiences with others facing similar circumstances, as explained by the following participant:

It would be important to share my experience with other people who may be experiencing the same issues, this would be helpful and allow to exercise one’s skills of listening to the other.

ID 11

The fourth theme highlights the need to increase one’s knowledge about their medical condition, obtain reliable information about chronic pain and treatment options, and directly consult experts in the field. The following are 2 exemplary quotes on this theme:

Since these are unknown diseases, there are no effective drugs, only supplements, it would be helpful to have a source of up-to-date information.

ID 7

An app where you can contact experts to ask for information, to receive support.

ID 10

### Potential Barriers to the Use of eHealth Tools

Finally, with regard to the potential barriers to using eHealth tools, the following four themes were identified: (1) *computer illiteracy* (n=7 quotes), (2) *negative effects or risks* (n=3 quotes), (3) *impersonal interaction* (n=2 quotes), and (4) *physical limitations* (n=2 quotes).

In general, the first theme concerns the perceived lack of technological skills or attitude and the reduced interest in learning how to use new technologies, sometimes citing advanced age or the belief that such digital devices are substantially useless in the treatment of pain as reasons. The following are some examples of quotes to support this theme:

I find technology difficult, I’m not capable, I never did bother to learn it.

ID 7

I’m not really into technology, it has never been my strong suit.

ID 14

Honestly, I’m not interested in learning to use technology... And then at my age...

ID 4

I'm not someone who believes in these fancy things. If you feel pain and it doesn’t go away, you keep it and that’s it.

ID 6

The second theme addresses concerns of older adults regarding potential risks or negative effects arising from the use of eHealth tools. On the one hand, these concerns pertain to the risk of developing technology addiction, as pointed out by the following interviewee:

Then you get used to it, you always look for more... you get addicted to that too.

ID 8

On the other hand, these risks might involve potential adverse effects. For example, 1 participant stated the following:

When you search for information on the internet, for example, sometimes it seems like you have more pain, you start to fantasize about having a serious pathology...

ID 3

The third theme explains the idea that interaction with a device cannot be considered as satisfactory as that with a real person (eg, health care professional), which is, instead, considered essential. In this regard, 1 participant stated the following:

I need to hear from someone, not technology... not for human issues like health care.

ID 9

In this sense, several participants considered the eHealth tools to be inadequate substitutes for in-person interaction, also expressing a clear preference for the latter:

I think talking to a person, to a doctor, is more helpful than looking at your cell phone. I prefer it...

ID 3

The fourth theme concerns physical limitations that could prevent the use of the technology, such as visual and hearing impairments, as reported by a participant:

I don’t even watch television, my hearing is bad, I understand little of what I hear, I get about 40% of what I listen to.

ID 13

**Figure 1:**
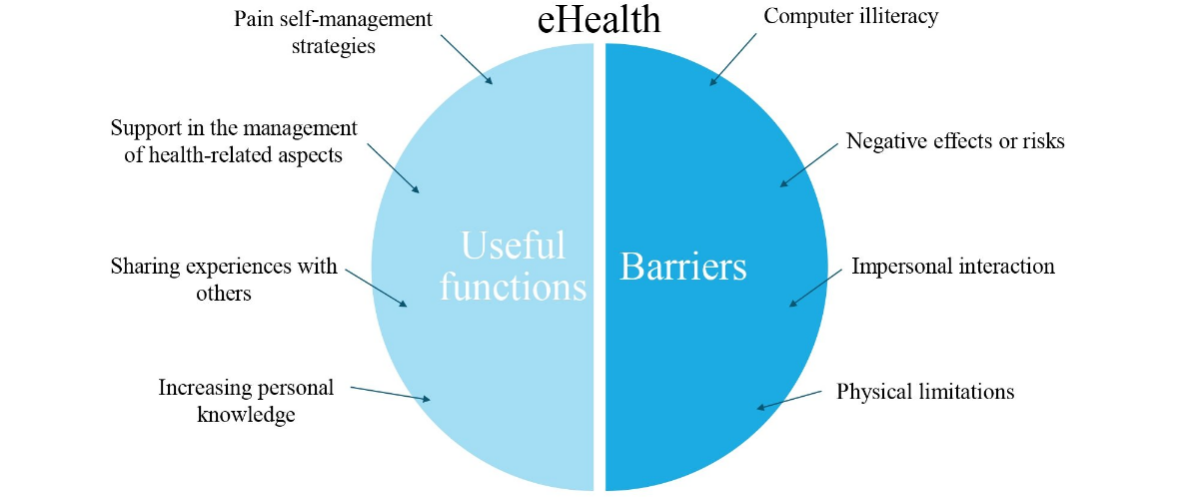
Potential useful functions and barriers to the use of eHealth solutions in the context of chronic noncancer pain management.

Figure 1 shows an overview of the themes related to the potential useful functions and the barriers to the use of eHealth solutions in the context of CNCP management.

## Discussion

### Principal Findings

This study aimed to explore perspectives and experiences of older adults (aged 65 to 80 years) with any type of CNCP regarding the strategies used to cope with chronic pain and the related perceived effectiveness; the types of digital technologies adopted in everyday life and the purposes of their use, including those related to health and pain management; the potential useful functions to be included in any eHealth solutions; and the potential barriers to use such tools in the context of chronic pain self-management. To the best of our knowledge, this is the first qualitative study to investigate the abovementioned aspects in a population of older adults with CNCP without focusing on a specific pain-related diagnosis but in the general context of a pain therapy outpatient center. Moreover, looking at the previous literature, the main research in this field has not been conducted in a Mediterranean country, such as Italy. This study thus enables insight into the specific features and needs of the Italian older population with chronic pain. This is of particular relevance considering that, as reported in the *Background* section, sociocultural factors may significantly affect how people perceive and manage well-being and health-related aspects, including the experience of chronic pain [11,29,43], along with the degree to which technology is accepted and used [31].

Regarding the first aim, the most frequently declared strategies to cope with chronic pain were coping self-statements, resting, task persistence, and guarding, while the least used strategies were relaxation and exercise or stretch. These findings are consistent with those found in 2 previous research carried out in the United States, targeting older adults with chronic pain, in facilities homes and community-based settings [44,45]. Interestingly, compared to those studies, we found greater use of resting as a strategy for managing pain, which was also qualitatively described as the most effective in terms of perceived pain reduction by the study sample. It must be noted that although a certain strategy was designated as the most effective, some participants were assigned a minimal score in terms of effectiveness in reducing pain. Similarly, although some strategies (eg, task persistence) were among the most widely used, they have been reported as effective by only a few participants. Overall, the results seem to show that in this study sample, the use of a specific strategy is not aimed at achieving a significant pain reduction but rather at obtaining some relief to be able to maintain their daily activities, often under the belief that pain will always be a part of their lives. As for this emerging attitude, the findings are consistent with the passive acceptance attitude that was found in older people with long-term pain conditions [8]. In this regard, it should be noted that this disposition may also have been influenced by the very long duration of chronic pain in our sample and by having experienced numerous medical treatments that were nevertheless unresolved. When faced with this sort of scenario, which often underlies a belief of total ineffectiveness and uselessness of any interventions, it is, therefore, of primary relevance to foster a more proactive attitude and simultaneously an increased self-efficacy to promote better chronic pain management, including the use of eHealth solutions.

Regarding the type of digital technologies used, all participants reported owning a mobile phone and using it independently, while only a few other digital tools were considered. This result is in line with international statistics indicating that the number of people aged >65 years who smartphones is increasing considerably, while for other modern technological devices (eg, tablets, computers, and smartwatches), there seems to still be a larger gap with younger age groups [46]. Especially following the acceleration induced by the COVID-19 pandemic, there has been an increasingly widespread use of technology, which also involved the older population. Indeed, some evidence showed higher levels of digital device adoption in daily life (eg, for entertainment, socialization, and health needs) by older adults already in the first stages of the COVID-19 pandemic [42,47,48]. Even in the Italian context, the frequency of use of digital technologies by the older population appears to have risen between 2019 and 2022 [49]. For example, the percentage of individuals who reported using a smartphone “often” or “always” increased by 16.3% (ie, from 37.7% in 2019 to 54% in 2022). This tendency is also supported by statistics from the European Commission, indicating that the percentage of Italian older people aged 65 to 74 years who have used the internet in the past 12 months increased from 44.6% in 2019 to 62.3% in 2023 [50]. Our results concerning the purposes of use in everyday life (eg, keeping in touch with others and leisure activities) also seem to point out in this direction. Despite this increase, it has to be considered that to date, a digital divide still remains with some northern European countries, where this percentage is significantly higher (eg, 97% in the Netherlands, 96.6% in Denmark and Luxembourg, and 94% in Sweden) [50].

Notably, none of the participants spontaneously indicated activities related in any way to health management, except for one who referred to using the internet to seek information about one’s medical condition or newly prescribed medications. Concerning the use of digital devices for health-related purposes, it is interesting to note that in our sample, these mostly involved very basic organizational activities (eg, booking medical visits and managing pharmaceutical prescriptions), and none of the participants reported the use of digital technologies for treatment purposes (eg, telemedicine) with respect to either their general health or CNCP condition. Although telemedicine interventions have also been implemented in Italy in the wake of the COVID-19 pandemic–induced acceleration [51], significant disparities remain in the country, with the telemedicine approaches (eg, televisiting, teleconsulting, and telemonitoring) offered to patients across the different Italian regions, varying in number from 1 (ie, telereferencing and televisiting in Basilicata Region and Autonomous Province of Bolzano, respectively) to 66 typologies (Lombardia Region) [52]. Moreover, according to the results of recent studies, a large proportion of Italian citizens are unaware of the availability of such digital health solutions [53], and especially among older adults living in remote areas of Italy, a digital health gap exists due to both infrastructural deficiencies and a lack of digital skills [54]. In light of the potential benefits offered by the digitization of the health care field, it would, therefore, be important to implement more large-scale initiatives aimed at raising awareness of the potentialities of digital technologies on a broad scale, involving both health care professionals as well as patients and their caregivers.

Consistent with the framework outlined here, several participants in our study described the experience of using eHealth tools as limited in terms of the number of activities performed or frequency. In addition, some older adults reported needing external assistance from informal caregivers in using digital devices or completely delegating to them the execution of health-related management activities, citing as the main explanation the lack of familiarity with the technology in general. This attitude may, to some extent, disincentive them from experiencing the use of eHealth tools on their own.

Nevertheless, in other cases, the experience of using such tools was defined as positive or helpful with respect to one’s purposes. Indeed, more than half of the respondents (10/18, 56%) were favorable about the potential usefulness of any eHealth tools specifically designed for chronic pain self-management, suggesting several potential useful functions to be included in such digital health solutions. More specifically, consistent with the findings of previous research [20,23], participants in this study highlighted the need to obtain updated and reliable information regarding their medical condition and available treatment options, including the option of contacting experts directly for questions or advice. In addition, there was a desire to improve pain self-management skills by learning new physical and psychological strategies. This highlights the need for targeted and diverse content to accommodate individual preferences. As observed in other studies [20,55], the inclusion in any eHealth solutions of features that allow remote interaction and sharing of experiences with others experimenting with a similar condition has been described as potentially helpful by several participants. This may also be beneficial in countering social isolation as a risk factor for poor well-being in older adults [56], which, especially in Mediterranean countries, seems to be one of the main factors affecting the psychological well-being older people aged 80 years and above [30].

Among the possible barriers to the use of eHealth solutions, just under half of the study participants (7/18, 40%) reported a lack of digital skills or technological attitude, resulting in a reduced sense of familiarity and trust in the potential of these tools for CNCP self-management. This is coherent with the evidence that older adults are less digitally literate than younger cohorts [57,58]. Even in previous research, this represents one of the main obstacles to the adoption of digital devices for health-related purposes [20,42]. Looking at the specific Italian sociocultural context, according to the Digital Economy and Society Index report of 2022 [59], Italy ranks 18th out of 27 European Union member states, and, to date, more than half of the Italian population does not have at least basic digital skills. If the analysis is extended to different age groups, the disparities widen even further: the percentage of Italian people who have at least basic digital skills stands at 42.2% in those aged 55 to 59 years and drops to 19.3% among those aged 65 to 74 years compared to 61.7% among young people aged 20 to 24 years [60]. Interestingly, according to a recent study that explored the digital divide of older adults living in peripherical areas of Italy, those with higher levels of education are more likely to use new digital technologies [54], and this could partly support our results because almost 80% (14/18) of our sample has a primary or middle school education level.

Among the potential barriers to the use of digital health tools, concerns about possible risks and negative effects were highlighted, such as the risk of developing a technological addiction or worsening one’s chronic pain condition. As previously mentioned, because these concerns may arise at least in part from misinformation, it might be useful to propose educational initiatives also aimed at promoting more accurate knowledge and thus greater awareness regarding the potential and the correct use of these tools in the health care context [61]. Regarding the physical limitations (eg, visual and hearing impairments) that could prevent the adoption of digital health devices, as pointed out in previous studies [20,23], it is crucial to attentively address design aspects to tailor the features of these tools to the age-related psychophysical needs of the older population (eg, provide adequate font size and screen brightness and the ability to select visual and auditory aids).

Finally, the lack of human interaction and the preference for an in-person relationship (ie, with health care providers) emerged in this study as possible deterrents to the adoption of eHealth solutions. This seems to be in line with previous evidence both internationally [62] and pertaining to the Italian population [53], which suggested a favorability for traditional visits over remote visiting modalities. In this regard, as pointed out by Bhattarai et al [23], it might be useful to support older people in the use of such tools by providing for the direct involvement of physicians and health care professionals and thus promoting such innovative modalities to be better integrated into the care process.

### Strengths and Limitations

This study has several strengths and limitations. The first strength is that it involved a diversified sample of Italian older adults, that is, including older people in the age range of 65 to 80 years experiencing a wide range of CNCP conditions, mainly of high-medium intensity and long-term duration, and referring to a clinical center for pain management. Although the upper age limit of the participants has been set to 80 years due to clinical and practical reasons (ie, physical and mental discomfort due to the time-consuming medical examination that preceded the evaluation), we have broadened the target population generally involved in these typologies of studies by including not only those aged 65 to 74 years but also a subgroup of people aged 75 to 80 years. This is noteworthy considering that people aged 75 to 80 years have traditionally been underrepresented in clinical research, despite the high comorbidity and presence of chronic pain [63].

In addition, a high percentage (18/23, 78%) of older individuals who visited the center during the recruitment period participated in the study, which helped reduce selection bias. As an additional strength, this is the only study conducted in Italy to have considered the older population with chronic pain. Considering the differences in the digital skills and pain perception across the culture and context, this represents a strength. The findings could inform the future development of eHealth solutions tailored to the specific needs and characteristics of the older Italian population with CNCP.

However, other limitations should be considered. First, we recruited a small sample size, although this was in line with other qualitative studies, and data saturation was achieved. This also precludes further considerations regarding the attitudes that emerged toward eHealth in relation to gender, age subgroups, or type of chronic pain. Therefore, future studies could focus on these aspects. Second, we enrolled participants among those already accessing health care services for pain management while not reaching out to individuals who might benefit and need eHealth solutions even more due to residing in geographically distant, poorly connected, or isolated areas.

### Conclusions

To conclude, this study contributed to integrating and extending the current literature on the potentiality and barriers of eHealth for chronic pain management among older adults with different types of CNCP. Being aware of the differences in pain perception and management and the level of digital skills according to the sociocultural contexts, the results of this study allowed us to explore perspectives and experiences about the eHealth solutions for coping with chronic pain in a sample of older adults in the context of an Italian pain therapy center.

Results have been discussed considering how health care services can be directed to promote the use of these solutions and improve the management of chronic pain in older people.

Overall, participants showed an attitude of resignation to their chronic pain condition. Moreover, the use of digital solutions for health and pain management purposes is still scarce, even considering the perception of a lack of digital skills or technological attitude, resulting in a reduced sense of familiarity and trust in the potential of these tools for CNCP self-management. However, older adults are able to identify potential in the adoption of such tools.

The findings of this study may inform the development of new digital health tools specifically targeted at the characteristics of the Italian older population with CNCP. They also provide insights into how these tools should be proposed for them to be useful and feasible, emphasizing, in this regard, the importance of enhancing self-efficacy in pain management and digital literacy among older adults.

To summarize, the need to foster educational initiatives on the actual potential and purposes of eHealth solutions emerged, addressing the specific needs and challenges encountered by older adults and eventually involving their formal and informal caregivers. At the same time, it is of paramount importance to increase public welfare policies aimed at enhancing the older population’s digital skills and consequently reducing the existing digital divide before introducing innovative eHealth solutions.

## Appendix

Multimedia Appendix 1 Semistructured interview guide.

## Abbreviations

Ben-SSC: Psychological Well-Being Questionnaire
CNCP: chronic noncancer pain
CPCI-I: Chronic Pain Coping Inventory−Italian version

## References

[1] Treede RD, Rief W, Barke A, Aziz Q, Bennett MI, Benoliel R, Cohen M, Evers S, Finnerup NB, First MB, Giamberardino MA, Kaasa S, Korwisi B, Kosek E, Lavand'homme P, Nicholas M, Perrot S, Scholz J, Schug S, Smith BH, Svensson P, Vlaeyen JW, Wang SJ (2019). Chronic pain as a symptom or a disease: the IASP Classification of Chronic Pain for the International Classification of Diseases (ICD-11). Pain.

[2] Sá KN, Moreira L, Baptista AF, Yeng LT, Teixeira MJ, Galhardoni R, de Andrade DC (2019). Prevalence of chronic pain in developing countries: systematic review and meta-analysis. Pain Rep.

[3] Cravello L, Di Santo S, Varrassi G, Benincasa D, Marchettini P, de Tommaso M, Shofany J, Assogna F, Perotta D, Palmer K, Paladini A, di Iulio F, Caltagirone C (2019). Chronic pain in the elderly with cognitive decline: a narrative review. Pain Ther.

[4] Stompór M, Grodzicki T, Stompór T, Wordliczek J, Dubiel M, Kurowska I (2019). Prevalence of chronic pain, particularly with neuropathic component, and its effect on overall functioning of elderly patients. Med Sci Monit.

[5] GBD 2021 Low Back Pain Collaborators (2023). Global, regional, and national burden of low back pain, 1990-2020, its attributable risk factors, and projections to 2050: a systematic analysis of the Global Burden of Disease Study 2021. Lancet Rheumatol.

[6] Chen CY, Verdoorn B (2020). Can pain management be safely optimized in older adults?. Mayo Clin Proc.

[7] Gillsjö C, Nässén K, Berglund M (2020). Suffering in silence: a qualitative study of older adults' experiences of living with long-term musculoskeletal pain at home. Eur J Ageing.

[8] Crowe M, Gillon D, Jordan J, McCall C (2017). Older peoples' strategies for coping with chronic non-malignant pain: a qualitative meta-synthesis. Int J Nurs Stud.

[9] Brunkert T, Simon M, Haslbeck J, Zúñiga F (2020). Who to talk to about my pain? A brief qualitative study on perception of pain and its management in Swiss nursing home residents. Pain Manag Nurs.

[10] Schoeb V, Misteli M, Kwan C, Wong CW, Kan MM, Opsommer E, Wong AY (2022). Experiences of community-dwelling older adults with chronic low back pain in Hong Kong and Switzerland - a qualitative study. Front Rehabil Sci.

[11] Wong TH, Lee KS, Lo SM, Kan MM, Kwan C, Opsommer E, Anwer S, Li H, Wong AY, Schoeb V (2023). Challenges, concerns, and experiences of community-dwelling older women with chronic low back pain-a qualitative study in Hong Kong, China. Healthcare (Basel).

[12] Ho LY (2019). A concept analysis of coping with chronic pain in older adults. Pain Manag Nurs.

[13] Yagiz JI, Goderis G (2022). The impact of the COVID-19 pandemic on eHealth use in the daily practice and life of Dutch-speaking general practitioners in Belgium: qualitative study with semistructured interviews. JMIR Form Res.

[14] Hadjiat Y, Arendt-Nielsen L (2023). Digital health in pain assessment, diagnosis, and management: overview and perspectives. Front Pain Res (Lausanne).

[15] De Lucia A, Perlini C, Chiarotto A, Pachera S, Pasini I, Del Piccolo L, Donisi V (2024). eHealth-integrated psychosocial and physical interventions for chronic pain in older adults: scoping review. J Med Internet Res.

[16] Mannheim I, Schwartz E, Xi W, Buttigieg SC, McDonnell-Naughton M, Wouters EJ, van Zaalen Y (2019). Inclusion of older adults in the research and design of digital technology. Int J Environ Res Public Health.

[17] Fischer B, Peine A, Östlund B (2020). The importance of user involvement: a systematic review of involving older users in technology design. Gerontologist.

[18] Stypinska J (2023). AI ageism: a critical roadmap for studying age discrimination and exclusion in digitalized societies. AI Soc.

[19] Gilbert RM (2022). Reimagining digital healthcare with a patient-centric approach: the role of user experience (UX) research. Front Digit Health.

[20] O'Reilly PM, Harney OM, Hogan MJ, Mitchell C, McGuire BE, Slattery B (2022). Chronic pain self-management in middle-aged and older adults: a collective intelligence approach to identifying barriers and user needs in eHealth interventions. Digit Health.

[21] Donisi V, Poli S, Mazzi MA, Gobbin F, Schena F, Del Piccolo L, Bigardi V, Gajofatto A, Rimondini M (2022). Promoting participatory research in chronicity: the ESPRIMO biopsychosocial intervention for young adults with multiple sclerosis. Front Psychol.

[22] Morote R, Las Hayas C, Izco-Basurko I, Anyan F, Fullaondo A, Donisi V, Zwiefka A, Gudmundsdottir DG, Ledertoug MM, Olafsdottir AS, Gabrielli S, Carbone S, Mazur I, Królicka-Deręgowska A, Knoop HH, Tange N, Kaldalóns IV, Jónsdóttir BJ, González Pinto A, Hjemdal O (2020). Co-creation and regional adaptation of a resilience-based universal whole-school program in five European regions. Eur Educ Res J.

[23] Bhattarai P, Newton-John TR, Phillips JL (2020). Apps for pain self-management of older people's arthritic pain, one size doesn't fit all: a qualitative study. Arch Gerontol Geriatr.

[24] Pearson J, Walsh N, Carter D, Koskela S, Hurley M (2016). Developing a web-based version of an exercise-based rehabilitation program for people with chronic knee and hip pain: a mixed methods study. JMIR Res Protoc.

[25] Fanning J, Brooks AK, Ip E, Nicklas BJ, Rejeski WJ (2018). A mobile health intervention to reduce pain and improve health (MORPH) in older adults with obesity: protocol for the MORPH trial. JMIR Res Protoc.

[26] Stamm O, Dahms R, Müller-Werdan U (2020). Virtual reality in pain therapy: a requirements analysis for older adults with chronic back pain. J Neuroeng Rehabil.

[27] Mace RA, Gates MV, Bullard B, Lester EG, Silverman IH, Quiroz YT, Vranceanu AM (2021). Development of a novel mind-body activity and pain management program for older adults with cognitive decline. Gerontologist.

[28] Janevic M, Robinson-Lane SG, Courser R, Brines E, Hassett AL (2022). A community health worker-led positive psychology intervention for African American older adults with chronic pain. Gerontologist.

[29] Rogger R, Bello C, Romero CS, Urman RD, Luedi MM, Filipovic MG (2023). Cultural framing and the impact on acute pain and pain services. Curr Pain Headache Rep.

[30] Castelletti C, Martín-María N, Cresswell-Smith J, Forsman AK, Nordmyr J, Ådnanes M, Donisi V, Amaddeo F, Miret M, Lara E (2021). Comprehending socio-relational factors of mental wellbeing in the oldest old within Nordic and Mediterranean countries. Ageing Soc.

[31] Cai Z, Fan X, Du J (2017). Gender and attitudes toward technology use: a meta-analysis. Comput Educ.

[32] Rinaldi EE, Strizzolo N (2022). Competenze digitali e finanziarie nel primo lockdown: l'impatto sul benessere finanziario degli anziani. Salute e Societa.

[33] Hennink MM, Kaiser BN, Marconi VC (2017). Code saturation versus meaning saturation: how many interviews are enough?. Qual Health Res.

[34] Nicholas M, Vlaeyen JW, Rief W, Barke A, Aziz Q, Benoliel R, Cohen M, Evers S, Giamberardino MA, Goebel A, Korwisi B, Perrot S, Svensson P, Wang SJ, Treede RD (2019). The IASP classification of chronic pain for ICD-11: chronic primary pain. Pain.

[35] De Beni R, Borella E, Carretti B, Marigo C, Nava L (2008). BAC. Benessere e Abilità Cognitive nell'età adulta e avanzata. Giunti O.S. Organizzazioni Speciali.

[36] Monticone M, Ferrante S, Giorgi I, Galandra C, Rocca B, Foti C (2013). Development of the Italian version of the 42-item chronic pain coping inventory, CPCI-I: cross-cultural adaptation, factor analysis, reliability and validity. Qual Life Res.

[37] Ryff CD (1989). Happiness is everything, or is it? Explorations on the meaning of psychological well-being. J Personal Soc Psychol.

[38] Braun V, Clarke V (2006). Using thematic analysis in psychology. Qual Res Psychol.

[39] Braun V, Clarke V (2019). Reflecting on reflexive thematic analysis. Qual Res Sport Exerc Health.

[40] Braun V, Clarke V (2020). One size fits all? What counts as quality practice in (reflexive) thematic analysis?. Qual Res Psychol.

[41] Lincoln YS, Guba EG (1985). Naturalistic Inquiry.

[42] Garcia Reyes EP, Kelly R, Buchanan G, Waycott J (2023). Understanding older adults' experiences with technologies for health self-management: interview study. JMIR Aging.

[43] Rodrigues-de-Souza DP, Palacios-Ceña D, Moro-Gutiérrez L, Camargo PR, Salvini TF, Alburquerque-Sendín F (2016). Socio-cultural factors and experience of chronic low back pain: a Spanish and Brazilian patients' perspective. A qualitative study. PLoS One.

[44] Ersek M, Turner JA, Kemp CA (2006). Use of the chronic pain coping inventory to assess older adults' pain coping strategies. J Pain.

[45] Molton I, Jensen MP, Ehde DM, Carter GT, Kraft G, Cardemas DD (2008). Coping with chronic pain among younger, middle-aged, and older adults living with neurological injury and disease. J Aging Health.

[46] Faverio M (2022). Share of those 65 and older who are tech users has grown in the past decade. Pew Research Center.

[47] Sixsmith A, Horst BR, Simeonov D, Mihailidis A (2022). Older people's use of digital technology during the COVID-19 pandemic. Bull Sci Technol Soc.

[48] Elimelech OC, Ferrante S, Josman N, Meyer S, Lunardini F, Gómez-Raja J, Galán C, Cáceres P, Sciama P, Gros M, Vurro C, Rosenblum S (2022). Technology use characteristics among older adults during the COVID-19 pandemic: a cross-cultural survey. Technol Soc.

[49] Melchior C (2022). Gli anziani e lo scarso utilizzo (e desiderio) di tecnologia digitale. Salute e Società.

[50] Statistics. Eurostat.

[51] Maria Cati M (2022). Innovation in the Italian national health system and the cost-reducing impact of e-health and telemedicine: the special case of the telemedicine project of the Casa Sollievo della Sofferenza Hospital (Apulia region)—when information and communication technology comes to the aid of the territory. Int J Appl Biol Pharm Technol.

[52] eHealth - Sanità digitale. Ministero della Salute.

[53] Gallè F, Oliva S, Covelli E, Del Casale A, Da Molin G, Liguori G, Orsi GB, Napoli C (2023). Introducing telemedicine in Italy: citizens' awareness of a new healthcare resource. Healthcare (Basel).

[54] Vainieri M, Vandelli A, Benvenuti SC, Bertarelli G (2023). Tracking the digital health gap in elderly: a study in Italian remote areas. Health Policy.

[55] Richardson JE, Lee JI, Nirenberg A, Reid MC (2018). The potential role for smartphones among older adults with chronic noncancer pain: a qualitative study. Pain Med.

[56] Nakagomi A, Tsuji T, Saito M, Ide K, Kondo K, Shiba K (2023). Social isolation and subsequent health and well-being in older adults: a longitudinal outcome-wide analysis. Soc Sci Med.

[57] van Deursen AJ (2020). Digital inequality during a pandemic: quantitative study of differences in COVID-19-related internet uses and outcomes among the general population. J Med Internet Res.

[58] Vercruyssen A, Schirmer W, Geerts N, Mortelmans D (2023). How “basic” is basic digital literacy for older adults? Insights from digital skills instructors. Front Educ.

[59] Italy in the digital economy and society index. European Commission.

[60] (2023). Competenze digitali e caratteristiche socio-culturali della popolazione: forti divari. ISTAT.

[61] Global strategy on digital health 2020-2025. World Health Organization.

[62] Wilson J, Heinsch M, Betts D, Booth D, Kay-Lambkin F (2021). Barriers and facilitators to the use of e-health by older adults: a scoping review. BMC Public Health.

[63] Ridda I, MacIntyre CR, Lindley RI, Tan TC (2010). Difficulties in recruiting older people in clinical trials: an examination of barriers and solutions. Vaccine.

