# Supplementary material for: Perspectives and Experiences on eHealth Solutions for Coping With Chronic Pain: Qualitative Study Among Older People Living With Chronic Pain
Source: JMIR Aging. 2024 Sep 5;7:e57196. doi: 10.2196/57196 (PMC11413546; doi:10.2196/57196)
Supplement: Multimedia Appendix 1 [file aging_v7i1e57196_app1.pdf]

Semi-structured interview guide

Coping strategies to manage chronic pain

1. Among the coping strategies mentioned in the questionnaire, is there a strategy that in your opinion works the most? How much do you think this strategy works and reduces pain from 1 to 10?
2. Is there any other strategy you use but are not among those mentioned?

Type of utilized digital technologies and main purposes of use

3. Do you own any technological devices? Which? Do you use these devices independently?
4. (If yes) For what purposes do you use such devices in your daily life?

Experiences regarding health-related purposes of use

5. Do you use these devices for purposes related to your health, including pain management?
6. (If yes) How? Can you give me some examples?
7. What is your experience in this regard?

Potential useful functions and barriers to use e-Health solutions for pain management

8. Do you think that e-Health solutions designed specifically to provide support in pain management would be helpful to you?
9. (If yes) In your opinion, how would this help you? What might be useful to include in such a digital device to help you manage pain? What factors do you think might interfere with the use of such tools?
10. (If not) Why? What factors do you think might interfere with the use of such tools?
